# Clipping the Wings of the Midwives

**Published:** 1896-05-09

**Authors:** 


### Clipping the Wings of the Midwives.

It is to be hoped that there are few people of common sense who will be found ready to admit that, in an age when knowledge is advancing and science is to be had for the asking at every corner, it is right and proper that women should be left to face all the dangers and perils of child-birth with no better assistance than is to be obtained from the dirty and ignorant attentions given, according to rule of thumb or local tradition, by the class of women immortalised in fiction under the name of "Mrs. Gamp." On this point all are agreed, but so soon as it is suggested that the only way out of the difficulty is to teach these people the elements of knowledge and of cleanliness, and to register those who are found fitted for their duties, the medical profession, or a part of it, is up in arms and protests that this means the creation of a new and ignorant class of practitioners and their introduction to a field of practice into which the legislature has already decided that people shall not enter until they are qualified also in the cognate sciences of medicine and of surgery.

Here, then, comes the real question which is of importance to the public. Are midwives to undertake the whole art and practice of midwifery, or are they to attend the simple cases only and send for the doctor when difficulties arise? The general public and ninety-nine midwives out of a hundred will say that this latter is all that they ask for, but there can be no doubt that there are a few who desire to undertake the full range of midwifery practice. To these we would say—Qualify as medical women, and as medical women you can operate as much as you like; but we hold that it would be utterly wrong, especially just at present, when numbers of women are taking the trouble to qualify themselves properly, that those who will not take that trouble should be allowed to enter by a back door into the same field of practice.

So think the doctors. There seems practical unanimity as to the necessity for banishing the old Mrs. Gamp, but of the young Miss Gamp, who is to take her place, they are suspicious; they think she is a flighty person, and they want, before letting her into the fold, to clip her wings.

With this object the Lancashire and Cheshire Branch of the British Medical Association have framed a Bill of their own, to which they have added an Appendix, which really is a modification of the regulations under which midwives work in Austria.

Now in regard to this Bill, and its Appendix, we need not hesitate to express our opinion that they are a considerable improvement both on the Bill framed by the British Medical Association and the one now before Parliament. Certainly it commits the absurdity of calling its midwives midwifery nurses. Women with only a three months' training clearly cannot be nurses; midwives they are, and, unless the English language is to be changed by Act of Parliament, midwives they will remain.

What the Bill does, however, is to make registration compulsory on all women who "habitually and for gain attend, or undertake to attend, women during labour," exceptions being made in case of emergency, when the services of a duly-qualified person are not available. From the public point of view this is the most important part of the Bill, because it will do what is required for the public safety, viz., put a stop to the present state of affairs. Of course, all midwives now in practice will be admitted to registration on favourable terms. The safety of the public, however, demands protection from something else besides crass ignorance. Protection from the danger of a little knowledge is equally necessary, and probably this can best be gained by the issue of some such code of regulations as is contained in the Appendix put forward by the Lancashire branch, into the precise details of which, however, we need not now enter. Doubtless it will clip the wings of the midwives, and if some of the more flighty of them should object, it will but show the necessity of such restrictions. What the public want is to provide safe attendance for poor people, and for people living in distant hamlets in cases of ordinary labour. This the Bill will provide, and if young ladies want to do operative work, let them qualify for it as their brothers have to do.

What is certain is that the days of legislation for the benefit of a particular profession are past. Neither for the doctors nor for the midwives will Parliament stir one inch. If Parliament is to be moved it must be by showing that it is to redress a public evil or do a public good, and neither of these can be said of such a Bill as is now before Parliament, which, while preventing a woman from using the name does nothing to prevent her from following the occupation of a midwife, and imposes no effectual restriction on the practice of surgical midwifery by those who may, after however short and imperfect a training, once get on the register, and no restrictions at all on those who choose to practice midwifery without taking any notice of the Act.

